# Quiet echo planar imaging for functional and diffusion MRI

**DOI:** 10.1002/mrm.26810

**Published:** 2017-06-26

**Authors:** Jana Hutter, Anthony N. Price, Lucilio Cordero‐Grande, Shaihan Malik, Giulio Ferrazzi, Andreia Gaspar, Emer J. Hughes, Daan Christiaens, Laura McCabe, Torben Schneider, Mary A. Rutherford, Joseph V. Hajnal

**Affiliations:** ^1^ Centre for the Developing Brain King's College London London UK; ^2^ Biomedical Engineering Department King's College London London UK; ^3^ Philips Healthcare Eindhoven Netherlands

**Keywords:** magnetic resonance imaging, diffusion MRI, functional MRI, sequence development, neuroimaging, fetal

## Abstract

**Purpose:**

To develop a purpose‐built quiet echo planar imaging capability for fetal functional and diffusion scans, for which acoustic considerations often compromise efficiency and resolution as well as angular/temporal coverage.

**Methods:**

The gradient waveforms in multiband‐accelerated single‐shot echo planar imaging sequences have been redesigned to minimize spectral content. This includes a sinusoidal read‐out with a single fundamental frequency, a constant phase encoding gradient, overlapping smoothed CAIPIRINHA blips, and a novel strategy to merge the crushers in diffusion MRI. These changes are then tuned in conjunction with the gradient system frequency response function.

**Results:**

Maintained image quality, SNR, and quantitative diffusion values while reducing acoustic noise up to 12 dB (A) is illustrated in two adult experiments. Fetal experiments in 10 subjects covering a range of parameters depict the adaptability and increased efficiency of quiet echo planar imaging.

**Conclusion:**

Purpose‐built for highly efficient multiband fetal echo planar imaging studies, the presented framework reduces acoustic noise for all echo planar imaging‐based sequences. Full optimization by tuning to the gradient frequency response functions allows for a maximally time‐efficient scan within safe limits. This allows ambitious in‐utero studies such as functional brain imaging with high spatial/temporal resolution and diffusion scans with high angular/spatial resolution to be run in a highly efficient manner at acceptable sound levels. Magn Reson Med 79:1447–1459, 2018. © 2017 The Authors Magnetic Resonance in Medicine published by Wiley Periodicals, Inc. on behalf of International Society for Magnetic Resonance in Medicine. This is an open access article under the terms of the Creative Commons Attribution License, which permits use, distribution and reproduction in any medium, provided the original work is properly cited.

## INTRODUCTION

While acoustic noise reduction is a general aim for MRI examinations to enhance patient comfort or to avoid unwanted activation in functional MRI (fMRI) studies 1, it is of particular importance for the success of fetal examinations. The vulnerability of the unborn human to excessive acoustic noise is postulated to contribute in the extreme case to high frequency hearing loss, shortened gestation, and decreased birth weight [for a review see reference 2]. While no incident involving MRI has been reported to date, and indeed retrospective studies of human subjects have shown no detectable long term effects of noise in fetal MRI 3, it puts a particular emphasis on adequate levels of protection for fetal MRI scans. Sound levels are typically expressed on a dB (A) scale, including a weighting to match the perceived relative loudness in the human ear (termed A‐weighting). A number of studies 4 state and discuss regulations for safe exposure, all stating the limits in dB (A). Studies evaluating the acoustic environment and level of protection provided by the maternal torso surrounding the fetus have been performed in sheep models 5, and an analogue has been studied in humans by placing a hydrophone placed in the fluid filled stomach of an adult male 6, which demonstrated a typical attenuation of 30 dB, although conditions in‐utero have been found to depend on the position of the fetus as well as the frequency of the sound 7. The thickness of the amniotic fluid layer contributes only marginally to sound attenuation in general 5.

External noise protection techniques for the fetus in‐utero are either not feasible (ear plugs, headphones) or not generally applicable (wrapping the mother's abdomen). Therefore, reducing the acoustic noise output of the scanner is particularly desirable in this subject group. In clinical practice, this is typically achieved by imposing constraints on gradient slew rate and amplitude. Control of acoustic output in this way is associated with decreased temporal and spatial resolution as well as limited scan efficiency for most sequences, it specifically restricts the performance of echo‐planar‐imaging (EPI) sequences.

Single‐shot EPI is widely used for advanced applications such as fMRI and diffusion MRI (dMRI), and as such is a key tool for connectome type studies 8 of the fetal brain and perhaps other applications involving pregnant subjects. The EPI readout critically relies on rapid switching of gradient polarity and fast gradient rises are commonly employed to shorten other EPI sequence components. Such fast single shot sequences provide an acoustic challenge that is not well addressed by simply de‐rating gradient performance. In addition, novel acquisition techniques such as multiband (MB) imaging 9 introduce extra gradient blips that further contribute to acoustic EPI noise. Finally, the emergence of novel analysis pipelines requiring high angular coverage for multi‐shell diffusion sequences and high temporal resolution for fMRI studies puts additional emphasis on the efficiency of the EPI acquisition. Obtaining this data in acceptable acquisitions times thus further motivates the use of highly accelerated and efficient EPI sequences.

The acoustic noise in the scanner is mainly generated by the gradient system, particularly if there is extremely rapid switching of gradient amplitude and polarity 10. The time varying currents, *I*, driven through the gradient coils by the gradient amplifiers lead to interactions with the static magnetic field *B* and thus to Lorentz forces ( F=I×B). These forces work against the coil stiffness and lead to the generation of sound pressure approximately proportional to the velocity of the coil former surface. These effects depend on the geometry and material properties of the individual scanner setup. As the resulting fluctuations and displacements are typically small, sound generation may be approximated as a linear system 11 that can then be characterized by frequency response functions ( ${\text{FRF}}_{i}(f)$) for each gradient coil i=x,y,z, where *t* is time and *f* is temporal frequency 11. The overall acoustic response $R_{i}(f)$ of a gradient coil running waveform $g_{i}(t)$ can then be expressed as:

(1) $$R_{i}(f)=\text{FT}(g_{i}(t))\cdot {\text{FRF}}_{i}(f).$$

This process is illustrated in Supporting Figure S1 for an example read‐out train (both read‐out and phase encoding gradients).

Ways to reduce the acoustic noise can be mainly split into two types, hardware based and software based sound reduction. The former include the use of sound‐attenuating materials 10, active noise cancellation 12, or destructive sound interference in gradient coil design through opposing Lorentz forces 13. For fetal MRI in a clinical setting with standard hardware, software based solutions provide a key approach to noise reduction.

Potential modifications include changes to sequence timings to avoid resonance peaks in the FRF 14, and adaptation of the gradient waveforms to limit gradient activity. Examples include using spiral gradients 15, trapezoids with specific slope to base ratio 16, remodeling of the waveforms using parallel imaging 17 and remodeled waveforms using splines 18 or sinusoidal transitions 19, 20, 21, 22. Due to the requirement of the high‐slew‐rate gradients for EPI, only the studies of Schmitter et al. 19 and Zapp et al. 20 of the above were successfully employed for single shot EPI scans.

Ott et al. 21 developed methods based on read‐out segmented dMRI scans, where 2‐ to 4‐fold sound reduction was achieved by increasing acquisition times by 27–54%.

For the requirements of fetal MRI, increases in scan time, and specifically in single shot EPI read‐out time, should be kept as small as possible due to expected high prevalence of fetal motion.

In addition, none of the above mentioned studies include MB acceleration 22 and the effect of the CAIPIRINHA shift 23 gradient blips 9, or the achievable sound reduction by careful design of these. Finally, the acoustic influence of the butterfly crushers placed around the refocusing radio‐frequency pulses used for dMRI, as well as possible solutions, have not been studied.

This article describes an efficient, MB accelerated single shot EPI acquisition scheme both for gradient echo (GE‐) EPI used for fMRI and diffusion‐weighted Spin Echo (dSE) EPI used for dMRI, which we term quiet EPI (QuEPI). Our approach reshapes all gradient waveforms and tunes the obtained more controlled spectral properties of the sequence elements to the specific scanner FRF. It results in an important reduction of the acoustic noise while maintaining high scanning efficiency.

It specifically includes

1. Methodological description of the gradient reshaping to narrow the spectral content.
2. Replacing CAIPIRINHA blips with overlapping sin‐shifted profiles.
3. A novel crusher strategy for dMRI with joint re‐winder and butterfly crushers aimed to keep echo time (TE) short despite the reduced crusher amplitudes.
4. Simulations to optimize the resulting flexible acquisition to the scanner specific FRF with consideration of the implications on the planned versus played out gradient waveforms.
5. In vivo experiments both in adults and fetuses to demonstrate the maintained image quality.
6. A small fetal diffusion study with 120 directions using QuEPI to illustrate its practical use.
7. Noise measurements to quantify achieved sound reduction.

## METHODS

Gradient FRFs can have complex characteristics that change rapidly with frequency, and this is the case for the Philips 3T Achieva scanner employed in the current study. To avoid high acoustic output, the spectra of the gradient waveforms, including all harmonics, should not have high intensity values that coincide with peaks in the FRF. This requirement motivates a core strategy that has been adopted in several studies, that is designing the spectral properties of the gradient profiles to make the resulting acoustic spectrum as narrow as possible and avoid generation of higher harmonics. It then becomes feasible to tune the gradient spectrum to match favorable local minima of the FRF.

To achieve the required spectral properties, the gradient waveforms for the imaging axes (read‐out, phase encoding and slice encoding including CAIPIRINHA blips) have been modified following three principles:

1. Design individual gradient objects to obtain single dominant frequencies reducing harmonic content (e.g., favor sinusoidal waveforms over trapezoids)
2. Expand and combine objects as far as possible given efficiency constraints to avoid rapid transitions in amplitude
3. Tune the main sequence frequencies to coincide with minima in the scanner specific FRF.

### EPI Gradients and Definitions

**Figure 1:**
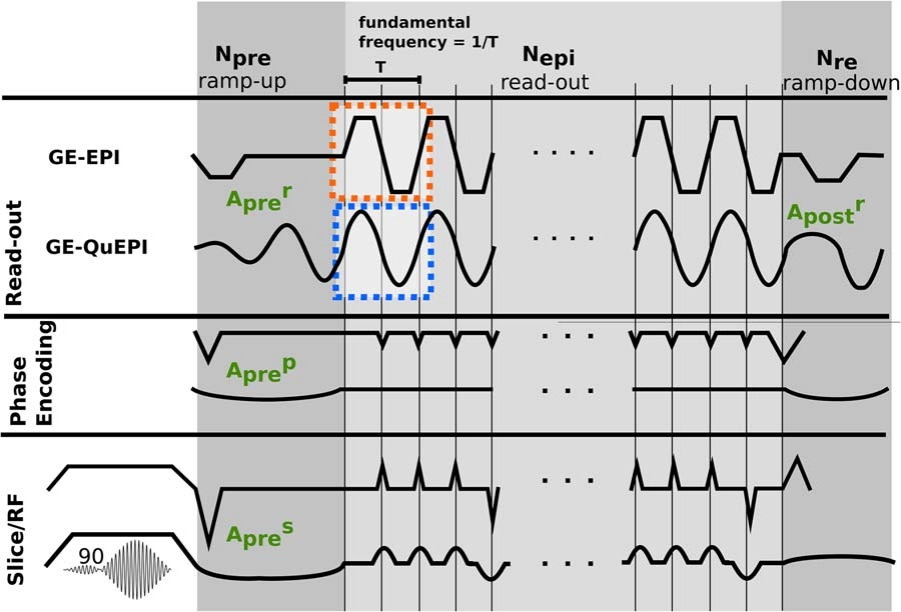
Sequence diagrams for conventional GE‐EPI and the proposed GE‐QuEPI sequence. Read‐out, Phase, and Slice gradient waveforms are shown separately for the ramp‐up, read‐out, and ramp‐down phases. Auxiliary gradients are marked in green.

Let the readout direction be *r*, phase *p*, and slice *s* in the following. A standard EPI read‐out train that encodes *e* lines of k‐space is composed of *e* gradient areas with switching sign to realize the required *k*‐space transversals in the *k_r_*‐direction as illustrated in Figure 1. These are interleaved with phase gradients (typically “blips”) for the variation in *k_p_*‐direction. The read and phase areas depend on the chosen resolution and the number of samples (see Appendix, Eqs. (A1) and (A2)).The CAIPIRINHA blips 23 required for optimized MB imaging, are parametrized by the CAIPIRINHA slice gap and the choice of the shift pattern as detailed in Appendix, Equation (A3). The timing of the EPI train is characterized by the echo spacing $t_{\text{echo}}$, which defines the fundamental frequency as $1/(2t_{\text{echo}})$. This is illustrated in Figure 1.

**Figure 2:**
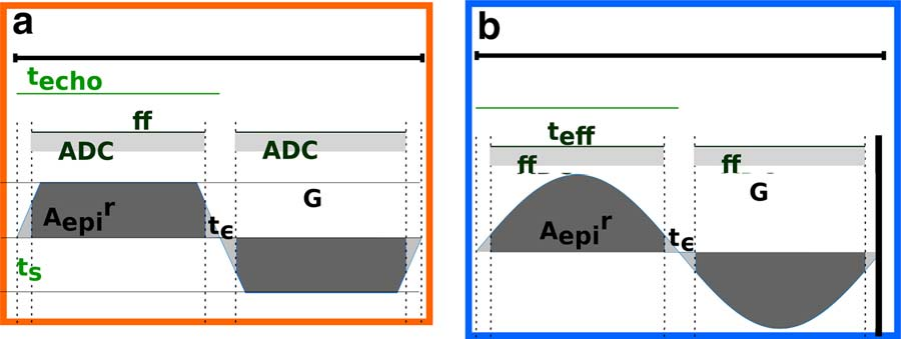
Timings for the read‐out axis are illustrated, including the period *T* (which encompasses two read‐outs), the effective sampling time $t_{\text{eff}}$ and the EPI waiting time $t_{\epsilon }$ as well as the slope time *t*_s_ for the trapezoids for (**a**) conventional GE‐EPI and (**b**) the proposed GE‐QuEPI sequence. The required read‐area $A_{\text{epi}}^{r}$ is shown as well as the adapted total areas for trapezoids and sinusoids leading to different gradient amplitudes.

Time gaps $t_{\epsilon }$ are introduced between successive sampling periods to control overlap with the phase gradients. These reduce the available sampling time to $t_{\text{eff}}=t_{\text{echo}}-t_{\epsilon }<t_{\text{echo}}$ and increase the required total area as illustrated in Figure 2a for conventional EPI and Figure 2b for the proposed QuEPI technique.

Finally, additional gradients required for navigation in k‐space include the read‐out, phase and slice pre‐winders of areas $A_{\text{pre}}^{r},\;A_{\text{pre}}^{p},\;A_{\text{pre}}^{s}$ as well as, in the case of spin echo EPI, spoiler gradients of area $A_{b}^{s}$ employed both around the refocusing radio‐frequency pulse, and area $A_{\text{post}}^{r}$ after the read‐out to de‐phase remaining magnetization. These are marked out in Figure 1.

### Efficiency and Acoustic Implications

To maximize efficiency and minimize echo time, all gradients are typically realized with trapezoids with maximal slew rate within the constraints given by hardware and peripheral nerve stimulation limits as shown in Figure 1. The acoustic implications are typically not a primary concern and are thus widely ignored. Typically, a gradient mode setting with maximal slew rate is employed for functional EPI studies to maximize efficiency. Due to the specific gradient strength requirements of dMRI, typically a gradient mode setting with a high Gmax is employed, which— depending on the employed scanner—limits the achievable slew rate.

To evaluate the acoustic output of this conventional read‐out, each trapezoid, described by time durations *a* and *b* with *a* > *b*, $r=\frac{b}{a}$ and amplitude *G^r^* (see Fig. 2a and Appendix, Eq. (A4)), can be visualized as a convolution of two rectangles, one equal to the temporal width of the trapezoid's full‐width‐half‐maximum $t_{\text{FWHM}}=\frac{a+b}{2}$ and the other to its slope time $t_{s}=\frac{a-b}{2}$, each with height *G*. The resulting spectrum then equals the product of two sinc functions of frequencies $\frac{1}{t_{\text{FWHM}}}$ and $\frac{1}{t_{s}}$. The trapezoids induce higher harmonics at multiples of the fundamental frequency $f=\frac{1}{2a}$.

The phase encoding and CAIPIRINHA blips contribute to a frequency at double the fundamental frequency set by the read‐out, with higher harmonics generally produced at multiples of this frequency. The exact spectrum depends on the blip shape properties (width, spacing, etc.) and, specifically for CAIPIRINHA blips, also on the chosen shift pattern, giving rise to a more complex frequency distribution. Finally, the relatively large areas required for crusher gradients and the need to keep these short in duration to promote temporal efficiency for the sequence as a whole typically leads to selection of fast rising, high‐amplitude gradient pulses, which contribute substantially to the acoustic output.

### Narrow Spectra Imaging Gradients

To narrow the spectral content, the read‐out was realized with sinusoids 19, 24 with a single frequency *f*, where the duration of a half sin wave corresponds to the echo spacing $t_{\text{echo}}$ sampled at time points *t_i_*, at increments of the dwell time of the scanner real‐time control system and with the corresponding amplitude taking the required acquisition pause for phase blips into account (time $t_{\epsilon }$ in Fig. 2a and Eq. (A5), Appendix). This frequency corresponds to the fundamental frequency.

The phase encoding blips were replaced by a constant low amplitude gradient 19 (Appendix, Eq. (A6)). This choice results in a narrowed spectrum as it reduces the intensity of the blip spectrum with its multiple higher harmonic peaks. The main spectral components lie within the low frequency range. This modification results in a sinusoidal k‐space sampling pattern as shown in Supporting Figure S3b.

**Figure 3:**
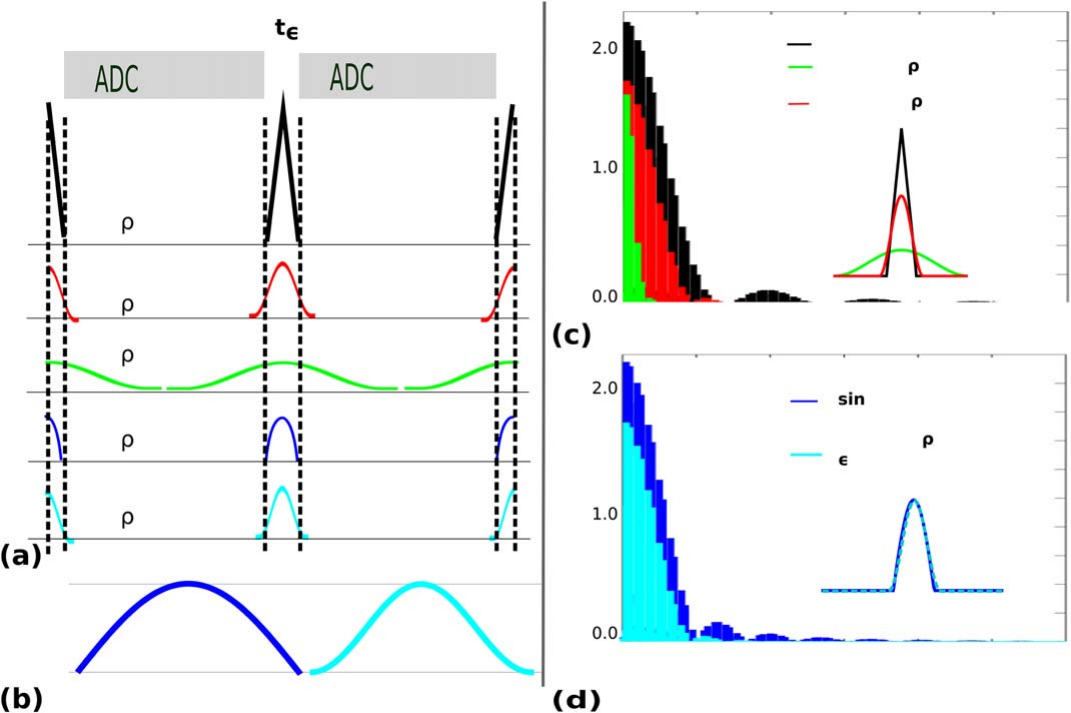
Illustration of the smoothed CAIPIRINHA blips. Five variants with matched area are considered: Conventional trapezoid (in this extreme case triangular) non‐overlapping blip (black), 10% overlapping sinWave object (red), 95% overlapping sinWave object (green), standard sinusoid (dark blue), and the proposed sinWave object (light blue). The objects are all shown in (**a**), the difference between sin and sinWave with matched amplitude and area in (**b**) and the corresponding spectra in (**c**) and (**d**), respectively.

The CAIPIRINHA blips required a different strategy to the primary phase encoding blips due to two differences. First, the chosen CAIPIRINHA aliasing pattern requires periodic gradient polarity reversals and varying blip gradient areas, precluding a continuous gradient, Second, these blips need to be fitted between data acquisition blocks with minimal overlap. This leads to a different optimum regarding spectral narrowing. Half sine waves sampled between [0 and *π*] are discontinuous at their start and end, leading to broader spectra, and were thus replaced in the QuEPI sequence by shifted full sine waves (called sinWave) sampled between [ $-\frac{\pi }{2}$ and $\frac{3\pi }{2}$] (see Fig. 3a,b and Appendix Eq. (A8) for exact definitions). The duration of the CAIPIRINHA blips *t* is often fixed to $t=t_{\epsilon }$ to avoid overlap with the read‐out. Here, the length of the objects can be individually controlled to allow an adjustable amount of overlap with the data acquisition as well as to account for the sharper rise of the last object for shift patterns with *s* > 2 (see Eq. (A3)). The overlap is parametrized in the following by ρ∈[0..1] with $t=t_{\epsilon }+\rho t_{\text{eff}}$. Thereby, ρ=0.95 corresponds to an almost complete overlap with the effective data sampling as illustrated in Figure 3a in green.

Figure 3c,d illustrate the spectral consequences of these design choices. Thereby, Figure 3c compares the relatively broad spectra with generated higher harmonics of the blips (depicted in black) with the proposed sinWave objects with an overlap factor of ρ=0.1 (red) and for illustrative purposes with ρ=0.95 (green). In Figure 3d, the differences between the two discussed sinWave objects is discussed. The standard sin object is shown in dark blue and the amplitude matched proposed sinWave object in light blue.

### Ancillary Imaging and Spoiler Gradients

Wherever possible, all remaining gradients were smoothed and extended while keeping time extensions to a minimum. Therefore, the read‐out module is constructed of three blocks all of which are constrained to conform to the timing of a single continuous sine wave to achieve maximally narrow frequency components (Fig. 1). The starting period has a flexible number of $N_{\text{pre}}$ cycles, followed by the required k‐space transversal in $N_{\text{epi}}$ cycles and the end period has $N_{\text{post}}$ cycles.

Within this framework, the read‐out pre‐winder is realized as a ramp‐up period for the $N_{\text{pre}}$ cycles. Both the phase and slice rewinder gradients are stretched to the corresponding time and implemented as sine waves. The phase pre‐winder was calculated to allow for the center of each complete read‐out traversal to be symmetric around the k‐space center. Where required, spoilers on all axes were converted to extended sinusoids matching the length of $N_{\text{post}}$ cycles. These changes are illustrated in Figure 1 and the calculations detailed in Appendix (Eq. (A9)).

The value of $N_{\text{pre}}$ directly impacts on the achievable echo time and together with $N_{\text{post}}$ also increases total acquisition time per slice, and so it is important to balance this cost with impact on the acoustics and harmonic generation of having rapid transitions. It is particularly important to minimize $N_{\text{pre}}$ as increasing echo time reduces signal strength, in this study $N_{\text{pre}}=2$.

### Crusher Strategy

Specifically for dMRI, significant acoustic contributions arise from the butterfly crushers around the refocusing radio‐frequency pulse. These dedicated crushers are required to provide sufficient dephasing to attenuate the Free Induction Decay (FID) resulting from imperfect refocusing. They can be omitted when the diffusion lobe areas before and after the 180° pulse fulfill this requirement, defining two cases for *b*‐values separated by a $b_{\text{min}}$. In our examples, $b_{\text{min}}$ was chosen as $50\;{s/\text{mm}}^{2}$.

**Figure 4:**
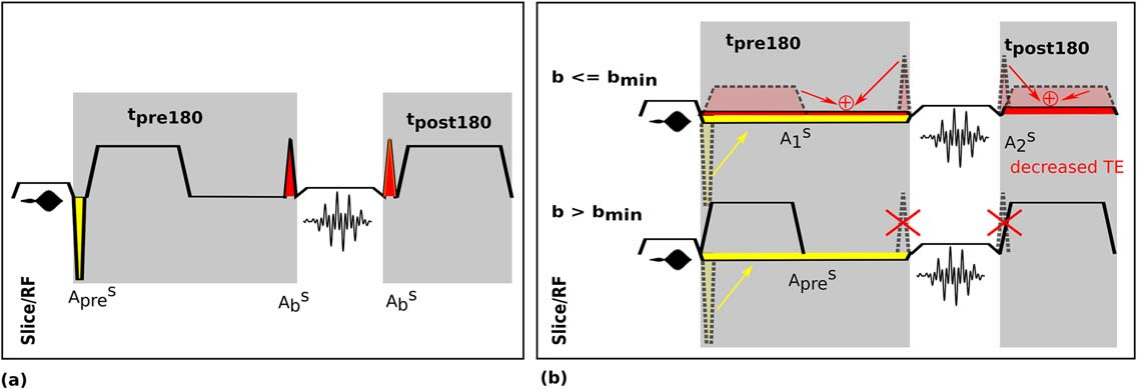
Illustration of the combined crusher strategy. The slice gradient waveforms are shown for (**a**) dSE‐EPI and (**b**) for the two cases ( $b\leq b_{\text{min}}$ and $b>b_{\text{min}}$) in dSE‐QuEPI. In our experiments, $b_{\text{min}}=50\;{s/\text{mm}}^{2}$. The required slice rewinder gradient area ( $A_{\text{pre}}^{s}$) is depicted in yellow and the butterfly crusher area ( $A_{b}^{s}$) in red. All modified gradient objects are depicted in gray dotted lines and colored arrows indicate how these get combined.

Omitting these dedicated crushers significantly reduces acoustic noise and can also decrease echo time. For QuEPI, this is achieved by combining the crusher pre‐refocusing gradient with the slice rewinder gradient (see Fig. 4) and stretching the combined lobe out in time, the post‐refocusing crusher is stretched in parallel to the second lobe of the diffusion preparation. For a slice rewinder gradient of area $A_{\text{pre}}^{s}$ and butterfly crushers of area $A_{b}^{s}$, the combined area $A_{1}^{s}=A_{\text{pre}}^{s}+A_{b}^{s}$ needs to be achieved before and the area $A_{2}^{s}=A_{b}^{s}$ after the refocusing pulse.

### Reconstruction

Our datasets contain both in‐plane and MB EPI accelerated acquisitions, so our general reconstruction method follows the extended SENSE framework proposed in Zhu et al. 25. Sinusoidal readout gradients combined with constant phase encode gradients results in exactly sinusoidal k‐space trajectories as illustrated in Supporting Figure S3a,b. The described trajectory combined with data sampling at fixed time increments results in the data not being on a regular grid in k‐space and thus there is a need for re‐gridding. Additionally, the continuous phase encoding gradient and its start at the very beginning of the sinusoidal read‐out waveform results in a shift of half a *k_y_* increment (corresponding to half a blip area) of the k‐space center as compared to conventional trajectories obtained using blips.

To limit the discrepancies between the conventional grid and the sample points in QuEPI, an additional half‐blip area is included into the phase encode pre‐winder to assure alignment of the k‐space centers. Furthermore, the outermost parts of the k‐space trajectory—and thus the parts differing most from the conventional grid—are not sampled and thus do not require further correction.

The required re‐gridding in the *k_x_* direction does not differ much from what is needed for conventional trapezoidal ramp‐sampling. The exact shape of the trapezoidal waveforms, especially the length of the plateau, depends on slew and Gmax settings and underlying limitations and thus requires adaptive re‐gridding.

QuEPI employs exactly the same vendor‐implemented *k_x_* gridding algorithm as any conventional EPI sequence. Regarding EPI ghost correction, different algorithms were tested in phantoms with only minor differences. Finally, a correction for non‐ideal gradient performance is included using a pre‐calibrated time delay.

### Practical Implementation and Testing

The sequence modifications described above were implemented for a standard 3T Philips Archieva TX system running R3.2 software. The sequence was validated in phantom experiments and tested using brain scans of two healthy adults. Two different protocol comparisons were performed for two separate purposes:

1. The scanner optimized sequence together with the QuEPI sequence were acquired on adult 1 (See sequence parameters in Supporting Table S1, Prot. 1 and Prot. 2) This protocol was used to compare image quality when operating under conditions differing maximally in acoustic noise.
2. The fetal dSE QuEPI sequence and an EPI sequence with matched fundamental readout frequency, in addition to the scanner optimized EPI sequence, all with matched repetition time (TR), TE and diffusion parameters were acquired on adult 2 (Prot. 3). Here, the three lower shells (*b*0, *b*400, and *b*1000) were used to allow for Signal to Noise Ratio (SNR) and quantitative diffusion parameter evaluation. The use of matched fundamental frequency readout waveforms allowed comparisons to be made using matched bandwidth with similar spatial distortions.

Both experiments needed to be performed on adult volunteers as the acoustic noise of the EPI version did not allow safe fetal operation. A standard 32‐channel head coil was used for signal reception.

### Acoustic Output Simulations

The acoustic output was simulated using custom‐made Matlab scripts fed with the scanner waveform output and the manufacturer supplied FRFs. The script (*acousticResponse.m*) is provided in the supplementary material and on the lab's GitHub page.^1^ The objective of these simulations was 2‐fold: generate a tool to assess the acoustic contributions from different object and parameter choices (such as fundamental frequencies, MB factors, and phase encoding choices) and illustrate the optimization and noise reduction achieved using QuEPI.

Finally, to conclude the evaluation for the technical performance, the waveforms for both dSE‐QuEPI and SE‐EPI were simulated using measured gradient impulse response functions 26 to assess the influence of inevitable non‐ideal performance of the gradient system and determine if correction was needed to achieve an accurate mapping to k‐space. The gradient impulse response functions include all aspects of the gradient response that contribute to the final true gradient field. This includes delays, eddy current effects and other issues such as the bandwidth of the gradient amplifiers and is valid so long as the gradient system can be treated as linear and time invariant.

### Acoustic Noise Measurements and Scanner FRFs

Due to the importance of the fundamental EPI frequency choice especially for any long dMRI and fMRI experiments, we empirically optimized it. We modified our QuEPI sequence further to have Cartesian trajectories available in the same framework with exactly matched fundamental frequency, and used this capability to measure the acoustic noise output of the basic GE‐ and SE‐QuEPI sequences for fundamental frequencies from 390 to 580 Hz in steps of 3 Hz. The vendor‐proposed solution for the case when no restrictions to slew rate/gradient strength are applied (resulting in slew 200 mT/m/s and Gmax 32 mT/m for fMRI and slew 100 mT/m/s and Gmax 64 mT/m for dMRI) was also measured. A further goal of this step is to validate the sound reduction achievable at the same frequency. The trapezoidal versions were always run at maximal slew rate but otherwise precisely matched to allow direct comparison.

The acoustic noise measurements were performed using an MR‐compatible Optoacoustics Fiber Optic Microphone (Optimic 1155) with a resolution of 0.1 dB, A and C weighting, with a sensor positioned at isocenter in the empty scanner bore, which is the typical location of the fetal head in a well‐planned examination. This experiment results in an optimal frequency choice which was then used for systematic testing of the acoustic output of both EPI and QuEPI versions including all described modifications, CAIPIRINHA blips and the different options for the slice rewinder and crushers.

### Fetal dSE‐QuEPI, GE‐EPI, and dMRI Experiments

Both acoustically optimized acquisitions were successfully used on 12 healthy pregnant volunteers using a 32‐channel cardiac coil for signal reception. Informed consent was obtained for each examination. Fat suppression was achieved using SPIR pre‐pulses 27 for all EPI acquisitions with the addition of reversed slice select gradients 28 during excitation and refocusing for SE‐QuEPI. This reversal of gradients was taken into account in the crusher strategy and did thus not lead to acoustic modifications. Image based shimming to second order optimized for the fetal head was employed.

QuEPI was applied for a range of in‐utero studies with several purposes, all sharing the need for an efficient, quiet EPI acquisition. Therefore, the employed parameters vary and the parameters are summarized in Supporting Table S1 for all subject data shown in the result section. These include both GE and SE EPI acquisitions, varying resolutions and acceleration strategies.

To illustrate the use of the developed protocols for efficient connectome studies high resolution multi‐shell acquisition, a high spatial and angular resolution dMRI protocol (Prot. 5) was acquired in six fetal subjects (subject 7–12). The parameters for this full dMRI protocol included 120 diffusion directions arranged across 6 *b*‐value shells [*b* = 0 (8), 400 (12), 1000 (20), 1400 (20), 1700 (30), 2000 s/mm^2^ (30)]. This 6‐shell approach was chosen over a more conventional 2–4 shell approach with higher angular resolution to allow comprehensive study of the fetal data signal content. The acquisition time for these datasets was 17 min. No additional averages were used, but increasing the number of angular samples with the *b*‐value provides oversampling in shells with more signal attenuation to reduce the noise in advanced models.

All data sets were corrected for motion and eddy‐current induced distortion with FSL 29, 30. Brain masks were obtained manually based on the mean *b* = 0 image. The multi‐shell HARDI data were subsequently decomposed into two sources using convex non‐negative spherical factorization 31, one orientation distribution function at spherical harmonic order Lmax = 4 and one isotropic volume fraction (Lmax = 0). This unsupervised source separation technique closely resembles multi‐tissue constrained spherical deconvolution 32 but avoids predefined response functions that are otherwise challenging to obtain in these fetal data sets.

## RESULTS

### Acoustic Output Simulations

**Figure 5:**
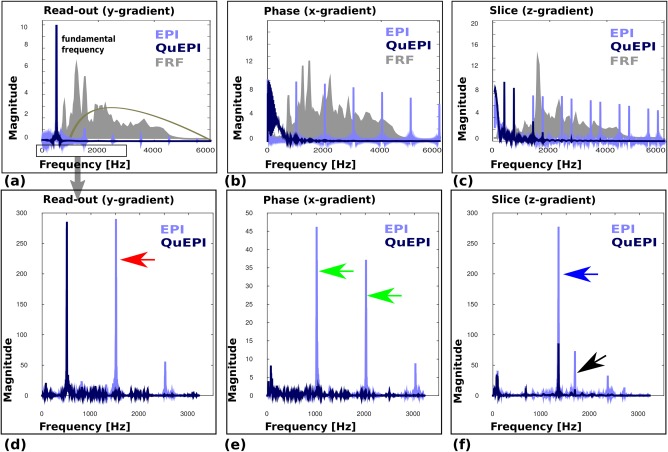
Simulated acoustic noise output for the dSE‐EPI and dSE‐QuePI. **a–c**: Gradient spectra for each sequence variant for each gradient axis together with their corresponding frequency response functions (FRFs). In (a), the A‐weighting dB curve has been added to indicate the frequencies of interest. **d–f**: Calculated acoustic responses for each gradient profile once the FRF is included. Gradient axes are specified as: Read (y‐axis), Phase (x‐axis), and Slice (z‐axis). Specific interesting frequencies are marked with arrows and discussed in the main text. Note the modified frequency scale in (d, e), which is used to focus on the dominant spectral components.

Simulation results for the gradient spectra from all gradient axes are shown in Figure 5a–c for dSE‐EPI and dSE‐QuEPI for a fundamental frequency of 507 Hz, illustrated because it produces low acoustic noise when harmonics are controlled (see below).

Whereas dSE‐EPI generates strong harmonics as well as the peak at the read‐out frequency of 507 Hz, only one dominant peak at the fundamental frequency can be seen in the spectra of the dSE‐QuEPI read‐out (Fig. 5a). Similarly for the gradient performing the phase encoding, no higher harmonics can be identified as can be seen in Figure 5b. Finally, while the slice gradients including slice rewinder and butterfly crusher for dSE‐EPI generate a wide spectrum, this is greatly reduced for the combined crusher strategy of dSE‐QuEPI (Fig. 5c). In the background of Figure 5a–c, the scanner‐specific FRF's are illustrated. In addition, the A‐weighting curve is shown in Figure 5a.

Figure 5d–f illustrates the calculated acoustic response spectra, obtained by multiplying the FRF with the simulated gradient spectra. The effects described above translate to much reduced harmonic spectral power for dSE‐QuEPI. In particular, the second harmonic for the read‐out at ∼1500 Hz (Fig. 5d, red arrow) and peaks from the phase encode gradient at ∼1000/2000 Hz (Fig. 5e, green arrows) in dSE‐EPI, which produces the highest peak in the acoustic response, are completely eliminated for dSE‐QuEPI (Fig. 5d,e). The z‐gradient (slice axis) acoustic response illustrates that the peak of the waveform spectrum around 1500 Hz is reduced (Fig. 5f, blue arrow), resulting in a more then 5‐fold decrease in the response spectrum at this frequency and the peak around 1800 Hz is avoided (Fig. 5f, black arrow). The FRFs on the corresponding gradient axes are shown in grey in the background of Figure 5a–c together with the A‐weighting curve (Fig. 5a only).

### Gradient Waveform Simulations

The simulation results from the various waveforms for trapezoidal and sinusoidal read‐out and phase show decreased deviations from the planned waveforms. This is evident especially in the onsets of the trapezoids and the blips compared to the nearly achieved sinusoidal and constant waveforms. The differences between planned and achieved waveforms illustrate the higher precision of the modified QuEPI gradient waveforms. The simulation results are shown in Supporting Figure S2. The required correction is, nevertheless, included into the gridding process as stated above.

### Acoustic Noise Measurements and Tuning to Scanner FRFs

**Figure 6:**
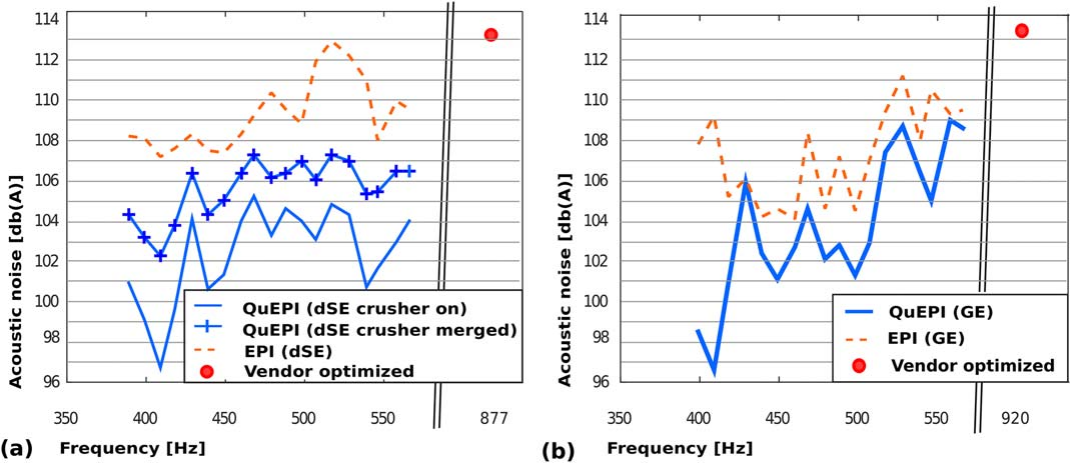
Acoustic output measurements for sequence variants as the readout fundamental frequency is varied between 380 and 600 Hz for both QuEPI and EPI. **a**: dSE: trapezoidal dSE‐EPI sequence (dotted line), dSE‐QuEPI sequence with sinusoidal slice rewinder (crossed line), and full dSE‐QuEPI sequence with the combined crusher strategy (solid line). **b**: GE: trapezoidal GE‐EPI sequence (dotted line) and GE‐QuEPI (solid line). In each graph, the scanner optimized, most efficient, settings (fundamental frequencies 877 Hz for dSE and 920 Hz for GE) are shown by a red dot. Note the break in frequency scale to accommodate this.

The greatest noise reduction is achieved by modifying the frequency content of the gradient waveforms on all three axes with respect to the FRF, in particular exploiting local minima. This applied in particular to the choice of the EPI fundamental frequency, for which a frequency sweep as described above was performed. The results of these measurements of fundamental frequency are shown in Figure 6 for both QuEPI and EPI. In each case, the scanner optimized solution is added at the frequency resulting from the vendor‐optimization (red dot).

For dSE, Figure 6a indicates the significantly reduced noise generation, approximately 8 dB (A) in the mean for all frequencies using QuEPI both with sinusoidal crushers (crossed line) and even more so with the combined crusher strategy (solid line). Compared to the scanner optimized most time efficient setting reached at 877 Hz, a reduction of up to 16 dB (A) is obtained with the quiet sequence operating at 410 Hz. For the scanner under test, local minima are predicted at 410, 440, 507, and 540 Hz for pure transverse acquisitions. Figure 6b shows the results for the GE sequence, which also demonstrate a general reduction in acoustic output, with maximal reductions achieved at 410, 507, and 540 Hz. While there is variation in the acoustic noise curves between dSE and GE, they share main characteristics such as the local minima around 410 and 450 Hz as well as a noticeable increase in noise around 580 Hz.

The choice of the fundamental frequency for dMRI and fMRI scans with high angular/temporal resolution was driven by seeking to achieve both low acoustic noise and high efficiency. Therefore, we selected the highest frequency, 507 Hz, and thus shortest echo spacing among the local minima in the noise measurements as a well suited compromise providing both high efficiency and low acoustic noise.

The results of the sound measurements are given in Table 1 for the chosen frequency of 507 Hz. For the GE experiments, QuEPI reduces acoustic output to 100.5 dB (A), from 113.4 dB (A) for the scanner optimized most efficient setting using an echo spacing of 0.54 ms or 106.7 dB (A) for a frequency—and thus echo spacing—matched trapezoidal acquisition. This constitutes reductions of 12.9 and 6.2 dB (A), respectively. The same hardware settings were chosen for slew and gradient amplitude for all frequencies for both QuEPI and EPI.

**Table 1:** Acoustic Noise Measurements for Different Settings of the dSE‐Echo Planar Imaging (EPI)/dSE‐Quiet Echo Planar Imaging (QuEPI) and GE‐EPI/GE‐QuEPI Sequences.

Sequence	Frequency (Hz)	Read‐out	Phase	Slice	CAIPIRINHA	Acoustic output dB (A)
**GE sequences**						
GE‐EPI	920	Trap	Blip	Trap	Blip	113.4
GE‐EPI	507	Trap	Blip	Trap	Blip	106.7
GE‐QuEPI	507	Sin	Cons	Sin	SinWave (0.1, 0.2)	100.4
**Evaluating CAIPIRINHA blips for GE sequences**						
GE‐QuEPI	507	Sin	Cons	Sin	Blip	101.0
GE‐QuEPI	507	Sin	Cons	Sin	SinWave (0.1, 0.2)	100.4
GE‐QuEPI	507	Sin	Cons	Sin	SinWave (0.95, 0.95)	99.6
**dSE sequences**						
dSE‐EPI	877	Trap	Blip	Trap	Blip	112.6
dSE‐EPI	507	Trap	Blip	Trap	Blip	111.8
dSE‐QuEPI	507	Sin	Cons	Comb	Sin (0.1, 0.2)	103.6
**Evaluating slice strategies for dSE sequences**						
dSE‐QuEPI	507	Sin	Cons	Trap	Sin (0.1, 0.2)	106.8
dSE‐QuEPI	507	Sin	Cons	Sin	Sin (0.1, 0.2)	105.8
dSE‐QuEPI	507	Sin	Cons	Comb	Sin (0.1, 0.2)	103.6

Measurements were taken using an optical microphone at the scanner isocenter in z‐direction at the height and approximate position of the fetal head.

Parameter legend: Blip, blip; Comb, combined; Cons, constant; Sin = sinusoidal; Sin(*ρ*_1_, *ρ*_2_) = sinusoidal; SinWave(*ρ*_1_, *ρ*_2_) = sinusoidal shifted; Trap(r) = trapezoidal (ratio).

While the combination of all elements leads to the stated reduction in sound, Table 1 gives in addition the sound measurements of two specific elements: for different realizations of CAIPIRINHA blips in the GE‐QuEPI sequence, a 1.4 dB (A) noise reduction is achieved using completely smoothed shifted sinusoidal CAIPIRINHA humps (sinWave) (see Fig. 3d) instead of blips. Similarly, for the tested dSE sequence with a *b*‐value of 1000, acoustic noise was reduced with QuEPI to 103.6 dB (A) compared to 112.6 dB (A) (most efficient setting at 877 Hz) or 111.8 dB (A) with matched echo spacing.

Finally, the different slice refocusing/spoiling strategies as discussed for dSE‐QuEPI were individually evaluated. The acoustic noise of the QuEPI sequence with trapezoidal slice rewinders and spoilers [106.8 dB (A)] could be decreased to 105.8 dB (A) using a sinusoidal rewinder gradient and even to 103.6 dB (A) when the combined spoiler and rewinder strategy is applied. In this concrete example, the combined crusher strategy also reduced the echo time by 1 ms.

Supporting Figure S4 shows the imaging results of the first adult‐experiment in which acoustically optimized QuEPI is compared to scanner optimized EPI, which entails operating at differing fundamental frequencies. Despite a 9 and 12.9 dB (A) reduction in generated acoustic noise, image quality is comparable. The increased distortion effects in QuEPI are attributable to the use of a lower bandwidth in the Phase Encode direction. This is due to a lower fundamental frequency (and thus echo‐spacing) in order to decrease acoustic noise. It was notable that the perceived sound quality was noticeably different, with a muted mellow tone for QuEPI compared to a harder more metallic sound for conventional EPI.

**Figure 7:**
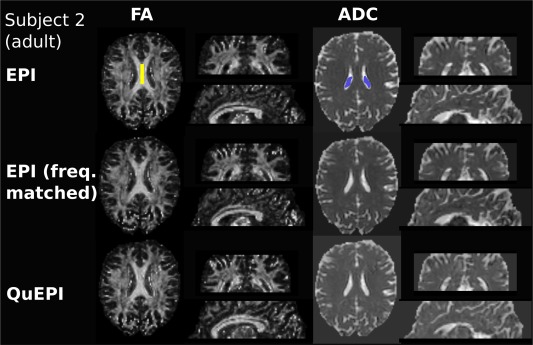
In vivo results from a healthy adult volunteer. fractional anisotropy and apparent diffusion coefficient results (Prot. 3, subject 2) obtained from diffusion data from conventional scanner‐optimized EPI, frequency matched EPI and optimized QuEPI (both run at a fundamental readout frequency of 507 Hz). The region of interests in corpus callosum and ventricles that were used to calculate the quantitative values in Table 2 are illustrated in yellow and blue. The acquired axial imagine planes are shown as well as reformatted coronal/sagittal views.

Calculated fractional anisotropy and apparent diffusion coefficient results are shown in Figure 7. These were obtained on a healthy adult (subject 2) with a conventional, scanner‐optimized EPI sequence, the full QuEPI sequence and an EPI sequence with matched fundamental frequency. All three versions were reconstructed using the standard scanner SENSE reconstruction and the same reference scan. The results from this second adult experiment are given in Table 2 showing similar SNR values and fractional anisotropy/apparent diffusion coefficient values in the corpus callosum and ventricles. The signal values were obtained by dividing the signal within the brain mask by the signal from the non‐zero voxels outside of the brain mask.

**Table 2:** Quantitative Comparison Between QuEPI, Conventional EPI and EPI With Matched Frequency.

		Corpus callosum	Ventricle
	Signal(brain)/ Signal (background)	Apparent diffusion coefficient/fractional anisotropy	Apparent diffusion coefficient/fractional anisotropy
**EPI**	19.7443	0.733 ± 0.159/0.836 ± 0.078	3.359 ± 0.602/0.230 ± 0.102
**QuEPI**	18.2027	0.735 ± 0.111/0.820 ± 0.068	3.325 ± 0.746/0.253 ± 0.094
**EPI (matched fre.)**	19.7456	0.754 ± 0.056/0.810 ± 0.058	3.466 ± 0.462/0.209 ± 0.068

The signal within the brain mask divided by signal in the background (excluding zeros) as a proxy for signal to noise/artefact ratio, as well as the fractional anisotropy and apparent diffusion coefficient values in regions of interest (shown in Fig. 7) are given.

**Figure 8:**
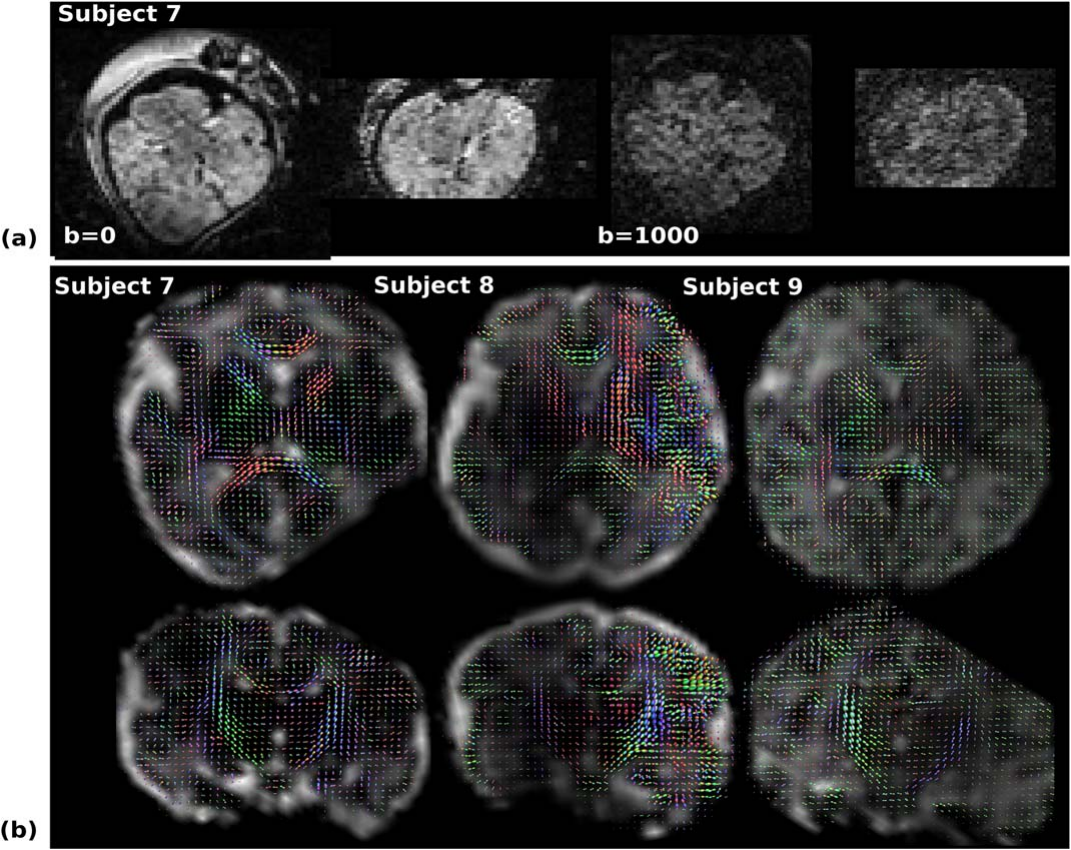
Illustrative results for subjects 7–9 from the 6‐shell HARDI protocol (Prot. 5) acquired using QuEPI. **a**: Example image data before processing (*b* = 0 and *b* = 1000) for subject 7 (GA 34 + 3 weeks) in axial (imaging plane) and reformatted coronal slices. **b**: Processed results for subjects 7–9 presented in transverse (top row) and coronal (bottom row) planes. Orientation distribution functions in each subject are color coded by direction in the scanner coordinate system and overlaid onto the isotropic volume fraction of a second component. The orientation distribution functions capture directional tissue structure in the corpus callosum and the corticospinal tract, as well as in cortical grey matter.

Results from the dMRI experiment using dSE‐QuEPI can be seen in Figure 8 illustrating the potential of QuEPI to acquire connectome data and the compatibility of QuEPI with advanced HARDI acquisition schemes. convex non‐negative spherical factorization recovered directional tissue structure in the orientation distribution functions, associated with white matter development in the corpus callosum and corticospinal tracts, as well as radial structure in cortical grey matter. The increased orientation distribution function amplitudes in the right frontal and parietal lobes in subject 8 is due to MRI intensity inhomogeneity (not corrected in post‐processing).

## DISCUSSION AND CONCLUSION

In this study, the use of gradient design with the guiding principal of minimizing generated spectral content of gradient waveforms in EPI sequences and then tuning the fundamental frequency to coincide with a minimum of the FRF of the gradient system has been explored as a means of decreasing acoustic noise. We term this approach QuEPI. QuEPI allows highly efficient fetal fMRI and dMRI acquisitions that are significantly less noisy than standard scanner EPI sequences.

Although there have been a number of previous strategies for reducing acoustic output from MRI sequences, these are either not feasible for EPI 18, 24, influence the applied acceleration factors or significantly increase acquisition time 17, 20. The previous study most closely suited for our application 19 was optimized for EPI techniques using sinusoidal gradients and constant Phase Encoding. It does, however, not include any optimization of CAIPIRINHA blips, butterfly crushers for dMRI as included in QuEPI and has, to the best of our knowledge, not be applied to fetal MRI studies.

Furthermore, most studies are focused on fMRI experiments only, whereas our approach decreases acoustic noise for all single shot EPI sequences which is a vital requirement for connectome type fetal studies. The dMRI‐specific modifications proposed in QuEPI, such as the combined crusher strategy, go beyond previously proposed alternations for single shot dMRI 21. We have also, for the first time, taken MB CAIPIRINHA blips fully into account. Also previous studies optimized the main frequency of the sequence to the acoustic output of scanner specific FRFs 14, 33, without attempting a complete redesign for all axes.

The proposed QuEPI framework is fully compatible with further acceleration techniques such as partial Fourier and SENSE in addition to MB, as was in fact demonstrated in the in vivo examples presented. A feature of QuEPI as implemented is its flexibility to optimize the read‐out frequency, diffusion gradients, and MB blips to the scanner specific FRF depending on the target acoustic noise output. The QuEPI approach provides a flexible platform for comprehensive fetal connectome examinations where high data rate with acceptable acoustic performance is needed, as well as other examinations where reducing acoustic noise output is important. The possibility to tune all elements of the sequence independently and synergistically to the scanner hardware allows to achieve optimal combinations of acoustic performance and efficiency. The range of experiments conducted for this study illustrates the high flexibility of the proposed framework.

The development of a merged crusher and rewinder gradient strategy, which switches structure as soon as the diffusion gradients intrinsically provide enough spoiling (i.e., when $b>b_{\text{min}}$) enables a significant reduction in sound level and can even result in a slight decrease in echo time. By taking MB acceleration into account and optimizing all gradients including CAIPIRINHA blips the framework provides a minimal sound solution to a key mode of accelerated acquisition that is increasingly used with EPI sequences. As demonstrated in Supporting Figure S4 and Table 2, the changes introduced in QuEPI to reduce acoustic noise did not result in changes in performance, other than those that must occur when echo time, readout bandwidth, and so forth are changed. This illustrates, that the use of QuEPI does not alter any calculated diffusion properties as a reduction in SNR could evoke 34.

The use of ramp‐up cycles slightly increases echo time, in our chosen examples by about 2 ms which might not be desired for certain acquisitions. But the flexible nature of the platform approach chosen here means, that the number of ramp‐up cycles can be chosen freely to balance additional echo time against decreases in acoustic noise.

The employed TE for fetal imaging is—apart from these slight ramp‐up times increases or decreases due to the crusher strategy—largely unchanged between QuEPI and conventional EPI. The minimum TE does depend on the chosen maximal *b*‐value/EPI factor in combination with the gradient mode setting, the slew rate, and maximal available amplitude. While all these parameters interact with each other, the use of QuEPI does not add extra limits.

Nevertheless, our study has the following limitations:

The use of the constant phase encoding gradient and the resulting shift of the frequency spectrum toward the low frequencies is advantageous in combination with the small weights in this range of the A‐weighting. However, the amplified low frequencies might in combination with different weightings be a less beneficial choice and further research would need to be done to explore this potential issue.

The use of conventional scanner gridding for the constant phase encoding performs well for the low accelerations factors as used in fetal imaging. This may, however, be a limitation for high acceleration factors and more elaborate methods as described for example in Ref. 19 might be beneficial.

The proposed simulations proved very useful in assessing and illustrating the effect of different gradient objects and parameter settings. They can, however, not replace the empirical data‐driven measurements for the fundamental frequency.

While oblique imaging planes are commonly used for fetal imaging, we employ pure scanner planes (purely transversal, sagittal, or coronal scan orientations) for our large fetal studies. This greatly simplifies, and so speeds up, planning and allows the acoustic noise contributions of individual gradient coil directions to be optimally fine tuned to the gradient system. The prevalence of fetal motion and the long scan durations needed for comprehensive diffusion and functional examinations often result in a change in the actually acquired fetal scan plane even when there is a precisely planned initial fetal brain geometry. These two factors support the concept of imaging in pure scanner planes combined with post‐processing reconstruction techniques 35. Fixed scan plane geometry also provides an added advantage of allowing key parameters, such as Field of View (FOV), to be standardized for all study subjects. Nevertheless, none of the presented concepts are limited to these choices. Knowledge of scanner specific FRFs allows optimal frequencies and noise peaks to be identified for any geometry. Once these are known, sequence parameters can be tuned to exploit the narrowed spectra to keep acoustic noise levels to a minimum. Future work could for example include real‐time feedback about increased acoustic noise for changed geometries.

The QuEPI approach can also be applied to conventional EPI sequences by formulating the optimization on the full acoustic response without also re‐designing the waveform structure. This more modest approach can still provide significant gains. Full inclusion of QuEPI combined with frequency tuning for low sound generation within scanner optimization code would be beneficial and will allow even more precise control of the acoustic sound output than is currently available. A possible further enhancement would be to optimize the diffusion gradients either by directly modifying the slew rate, or by adapting the trapezoidal ratio to help eliminate or control certain harmonic frequencies.

Previous studies have targeted general improvements in patient comfort or decreased acoustic stimulation for fMRI experiments. The QuEPI framework developed in this study was purpose‐built not only to enhance acceptability for fetal scanning, but also to render it efficient for connectome style prolonged EPI based studies. Previously such studies required to compromise in either efficiency, the number of possible diffusion directions sampled or number of complete volumes sampled. The shown fetal in vivo diffusion data illustrates the versatility and efficiency of QuEPI. It allows to run ambitious studies aimed to reveal for example the microstructural connectivity in‐utero by reducing fetal MRI scan time while allowing high angular or temporal coverage in an acoustically optimized and safe setup. This will greatly improve future functional and diffusion studies.

## Supporting information


**Table S1**. Imaging Protocols Used for the Experiments Shown in the Results Section. Subjects 1–2 Are Adult Volunteers, Subject 3–12 Are Fetal Volunteers. Abbreviations Used: MB, Multiband; PF, Partial Fourier; Res.: Resolution; SB: Singleband
**Fig. S1**. Acoustic simulation for a conventional EPI readout gradient with trapezoids for the case of flat top to base ratio of *r* = 0.8 and fundamental frequency of f=500 Hz. Left box: the gradient waveform *g*(*t*); Right box: corresponding frequency spectrum ( FT(g(f))), the gradient system acoustic frequency response function (FRF(*f*)) and, finally, the resulting acoustic output *R*(*f*). These results were generated using the Supporting script *acousticResponse.m*.
**Fig. S2**. Simulations of gradient performance using measured gradient impulse response functions. Planned and achieved waveforms for a single EPI readout lobe for (**a**) EPI and (**b**) QuEPI. Native waveforms and differences between planned and achieved waveforms are shown for all axes for GE‐EPI in (**c**) and differences only for GE‐QuEPI in (**d**).
**Fig. S3**. Illustration of gradient waveforms and resulting k‐space trajectories for EPI and QuEPI. **a**: Sequence details for EPI (left) and QuEPI (right). For QuEPI both the nonshifted and the half‐blip corrected version are shown. **b**: Resulting k‐space trajectories for all three mentioned versions together with the k‐space center (in *k_*x*_* direction) in orange.
**Fig. S4**. In vivo results from a healthy adult volunteer. Imaging data from EPI and QuEPI sequences (Prot. 1/2, subject 1) for both SE and GE sequences. The acquired axial imagine planes are shown as well as reformatted coronal/sagittal views.
**Script *acousticResponse.m***. Matlab script developed to simulate the acoustic response of pulse sequences. Requires the waveform on all three axes as well as the scanner individual FRFs as input.

## Footnotes

1
https://github.com/mriphysics.

## Notation and Abbreviations

Be *v_x_*, *v_y_* the resolution in *x* and *y* and $N_{\text{samples}}$ the number of samples. For CAIPIRINHA, be c[mm] the slice gap and the choice of the shift pattern described by *s* (Fig. 3b). All areas and gradient strength on the read‐out axis will be denoted with superscript *r*, on the phase encoding axis with *p* and on the slice axis with *s*.

Areas belonging to the EPI train will be denoted with subscript epi, objects pre‐EPI train with pre and post‐EPI train with post. All areas are in $\left[\frac{\text{mTms}}{\text{mm}}\right]$, gradient strength in $\left[\frac{\text{mT}}{\text{mm}}\right]$.

### EPI Train Areas

The read‐out, phase, and alternating CAIPIRINHA object areas are calculated as

(A1) $$A_{\text{epi}}^{r}=\Delta k_{r}N_{\text{samples}}=\frac{1}{\gamma v_{r}}$$

(A2) $$A_{\text{epi}}^{p}=\frac{1}{\gamma v_{p}}$$

(A3) $$A_{\text{caipi}1}^{s}(s,c)=\underset{\times s-1}{\underset{︸}{\frac{1}{s(\gamma c)}}}\;\;\ldots \;\;A_{\text{caipi}}^{s}(s,c)=\frac{-(s-1)}{s(\gamma c)}.$$

### EPI Train Amplitudes

The required gradient amplitudes equal

(A4) $$G^{r,\text{trap}}(A_{R},t_{\epsilon },t_{\text{echo}},b)=\{\begin{array}{c} \frac{A_{\text{epi}}^{r}}{\left(t_{\text{echo}}+b)/2-t_{\epsilon }^{2}/(t_{\text{echo}}-b\right)}\;\text{for}\;\;b<t_{\text{echo}}-t_{\epsilon }\;\;\text{and}\\ \frac{A_{\text{epi}}^{r}}{t_{\text{echo}}-t_{\epsilon }^{2}}\;\text{for}\;\;b\geq t_{\text{echo}}-t_{\epsilon }. \end{array}$$

(A5) $$G^{r,\text{sin}}(A_{\text{epi}}^{r},t_{\epsilon },t_{\text{echo}})=\frac{A_{\text{epi}}^{r}\pi }{1/2f\cos(\frac{\pi }{2}\frac{\left(1-(t_{\text{echo}}-t_{\epsilon })\right)}{\left(1/2f\right)})}\;\;\;\text{with}\;\;\;f=\frac{1}{2t_{\text{echo}}}.$$

(A6) $$G^{p,\text{const}}(A_{\text{epi}}^{p},e,f)=\frac{A_{\text{epi}}^{p}(e-1)}{e/(2f)}\pi f.$$

(A7) $$G^{s,\text{sin}}(a,f,t)=a\;\sin(2\pi ft)$$

(A8) $$G^{s,\text{sinWave}}(a,f,t)=a\;\sin(2\pi 2f(t-\frac{\pi }{2}))+a$$

### Auxiliary Gradients

The use of $N_{\text{pre}}$ cycles results in a pre‐EPI time of $\frac{N_{\text{pre}}}{2f}$ and thus a frequency of $f_{\text{pre}}=\frac{f}{N_{\text{pre}}}$ for stretched sinWave objects.

The requirement for the ramp‐up gradient equals

(A9) $$\int_{0}^{\frac{N_{\text{pre}}}{2f}}G(t)\text{sin}(-t)dt=\frac{A_{\text{epi}}^{r}}{2}$$

(A10) $$\Leftrightarrow G_{\text{pre}}^{r}(t,A_{\text{epi}}^{r},f,N_{\text{pre}})=\frac{2A_{\text{epi}}^{r,\text{sino}}\pi f^{2}t}{N_{\text{pre}}}.$$

The read spoiler is calculated as

(A11) $$A_{\text{pre}}^{r}=\frac{A_{\text{epi}}^{r}}{2}.$$

The phase encoding spoiler is calculated as

(A12) $$A_{\text{pre}}^{p}=\frac{eA_{\text{epi}}^{p}}{2},$$

but was limited to $\frac{e-0.5}{2}A_{P}$ to allow for the center of each complete read‐out traversal to be symmetric around the k‐space center.
