# Supplementary material for: Quiet echo planar imaging for functional and diffusion MRI
Source: Magn Reson Med. 2017 Jun 26;79(3):1447–59. doi: 10.1002/mrm.26810 (PMC5836719; doi:10.1002/mrm.26810)
Supplement: Supplementary file 1 — Table S1. Imaging Protocols Used for the Experiments Shown in the Results Section. Subjects 1–2 Are Adult Volunteers, Subject 3–12 Are Fetal Volunteers. Abbreviations Used: MB, Multiband; PF, Partial Fourier; Res.: Resolution; SB: Singleband Fig. S1. Acoustic simulation for a conventional EPI readout gradient with trapezoids for the case of flat top to base ratio of r = 0.8 and fundamental frequency of f=500 Hz. Left box: the gradient waveform g(t); Right box: corresponding frequency spectrum ( FT(g(f))), the gradient system acoustic frequency response function (FRF(f)) and, finally, the resulting acoustic output R(f). These results were generated using the Supporting script acousticResponse.m. Fig. S2. Simulations of gradient performance using measured gradient impulse response functions. Planned and achieved waveforms for a single EPI readout lobe for (a) EPI and (b) QuEPI. Native waveforms and differences between planned and achieved waveforms are shown for all axes for GE‐EPI in (c) and differences only for GE‐QuEPI in (d). Fig. S3. Illustration of gradient waveforms and resulting k‐space trajectories for EPI and QuEPI. a: Sequence details for EPI (left) and QuEPI (right). For QuEPI both the nonshifted and the half‐blip corrected version are shown. b: Resulting k‐space trajectories for all three mentioned versions together with the k‐space center (in kx direction) in orange. Fig. S4. In vivo results from a healthy adult volunteer. Imaging data from EPI and QuEPI sequences (Prot. 1/2, subject 1) for both SE and GE sequences. The acquired axial imagine planes are shown as well as reformatted coronal/sagittal views. Script acousticResponse.m. Matlab script developed to simulate the acoustic response of pulse sequences. Requires the waveform on all three axes as well as the scanner individual FRFs as input. [file MRM-79-1447-s001.pdf]

Supporting Figure S1:

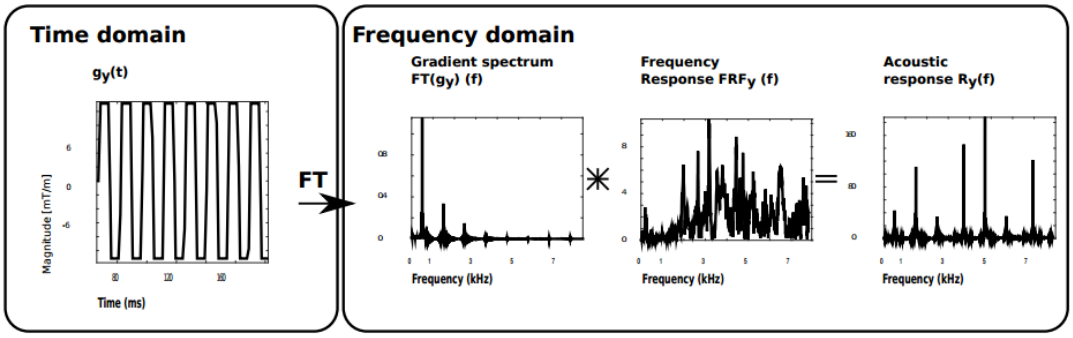

Supporting Figure S1: Acoustic simulation for a conventional EPI readout gradient with trapezoids for the case of flat top to base ratio of $r = 0.8$ and fundamental frequency of $f = 500Hz$. Left box: The gradient waveform $g(t)$; Right box: Corresponding frequency spectrum $(FT(g(f)))$, the gradient system acoustic frequency response function $(FRF(f))$ and, finally, the resulting acoustic output $R(f)$. These results were generated using the supporting script acousticResponse.m.

Supporting Figure S2:

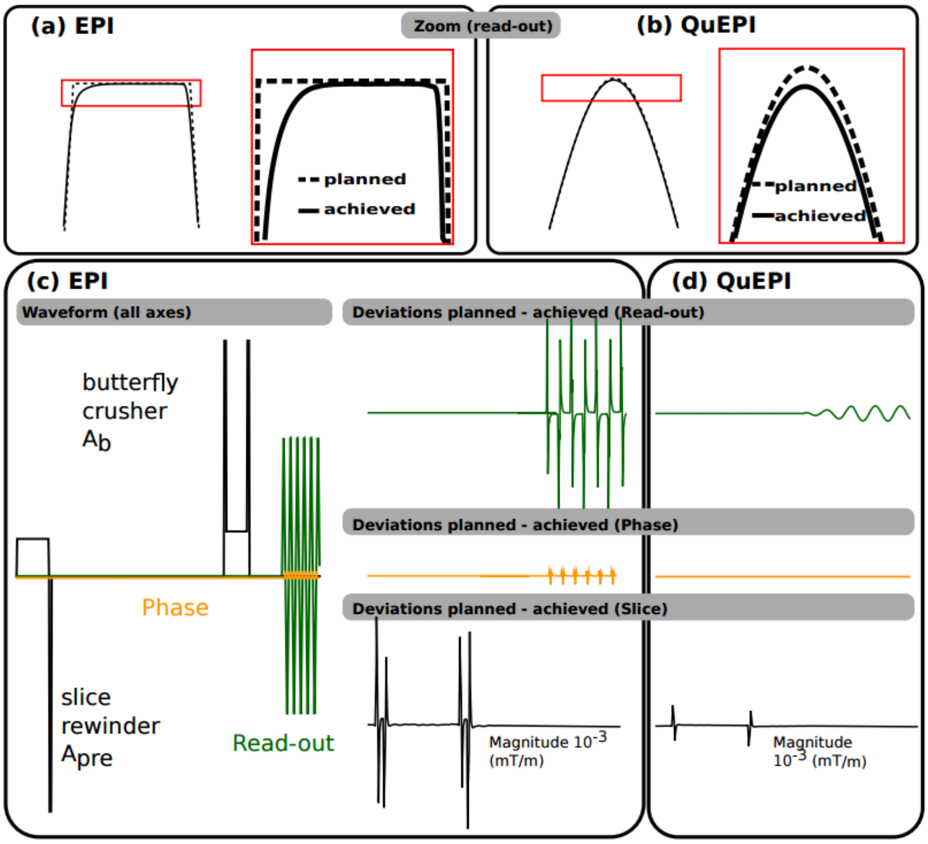

Supporting Figure S2: Simulations of gradient performance using measured Gradient Impulse Response Functions. Planned and achieved waveforms for a single EPI readout lobe for (a) EPI and (b) QuEPI. Native waveforms and differences between planned and achieved waveforms are shown for all axes for GE-EPI in (c) and differences only for GE-QuEPI in (d).

Supporting Figure S3:

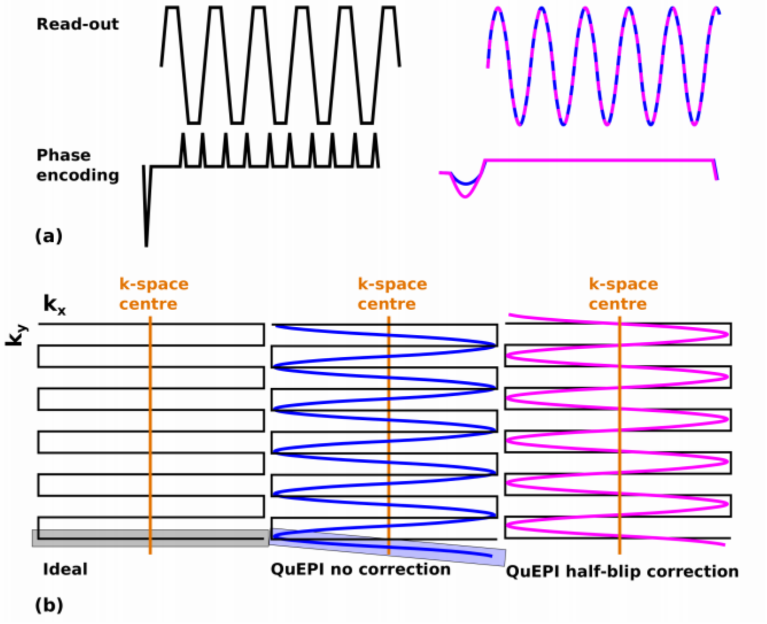

Supporting Figure S3: Illustration of gradient waveforms and resulting k-space trajectories for EPI and QuEPI. (a) Sequence details for EPI (left) and QuEPI (right). For QuEPI both the non-shifted and the half-blip corrected version are shown. (b) Resulting k-space trajectories for all three mentioned versions together with the k-space center (in k_x direction) in orange.

Supporting Figure S4:

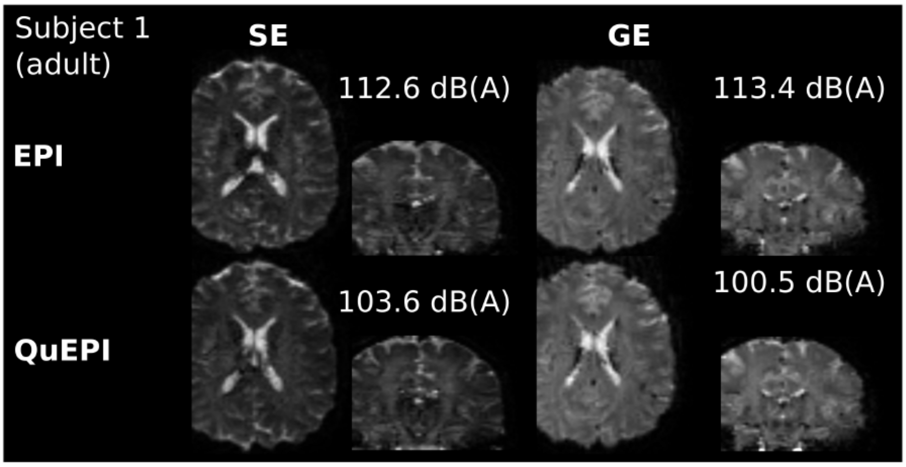

Supporting Figure S4: In-vivo results from a healthy adult volunteer. Imaging data from EPI and QuEPI sequences (Prot. 1/2, subject 1) for both SE and GE sequences. The acquired axial imagine planes are shown as well as reformatted coronal/sagittal views.

Supporting Table 1:

	Seq.	Method	Timing & Geometry	Acceleration
**Prot. 1** Subj. 1	GE	QuEPI/EPI	TR 5.0s, TE 50ms, Res. 2.5 mm^3	MB4, shift 3, Sense 1.4, PF 0.827
**Prot. 2** Subj. 1	SE	QuEPI/EPI	TR 5.0s, TE 100ms, Res. 2.5 mm^3	MB4, shift 3, Sense 1.4, PF 0.827
**Prot. 3** Subj. 2	SE	QuEPI/EPI	TR 8.5s, TE 153ms, Res. 2.2mm^3	SB, Sense 1.8, PF 1.0
**Prot. 4** Subj. 3-6	SE	QuEPI	TR 10.0s, TE 146.2ms, Res. 2.0mm^3	MB2, Sense 1.8, PF 0.85
**Prot. 5** Subj. 7-12	SE	QuEPI	TR 8.5s, TE 153ms, Res. 2.2mm^3	SB, Sense 1.8, PF 1.0

Supporting Table S1: Imaging protocols used for the experiments shown in the results section. Subjects 1-2 are adult volunteers, subject 3-12 are fetal volunteers. Abbreviations used: Res.: Resolution, PF: Partial Fourier, MB: Multiband, SB: Singleband.

Supporting Script S1:

```
Function acousticResponse(N,GR_DWELL,gr,sty,dirA,first,timeStart,figNr)
%INPUT:     N        :   number of sampling points
%           GR_DWELL:   dwell time of the system (temporal distance of
%           samples)
%           sty      :   style used for legends
%           dirA     :   Array used for the scan directions
%           (Read/Phase/Slice)
%           first    :   first example -> used for plotting
%           timeStart:  Start time of sequence (relevant as slice gradients
%           typically before 0)
%           figNr    :   used to plot into right figure
%
%           TO BE SUPPLIED IN ADDITION: Scanner individual transfer
%           function (mp_spl_trf_xyz.txt)

 fs=(1/GR_DWELL) ; %sampling frequency in 1 / s

   [B,A]=adsgn(fs);
   [H,W]=aspec(B,A,fs);

   load('mp_spl_trf_x.dat');  % supply frequency response function here
   load('mp_spl_trf_y.dat');
   load('mp_spl_trf_z.dat');

   irfh(dirA(1),:,:)= mp_spl_trf_x;
   irfh(dirA(2),:,:)= mp_spl_trf_y;
   irfh(dirA(3),:,:)= mp_spl_trf_z;

   fs_i = irfh(1,2,1)-irfh(1,1,1);
   irf(:,:,1)=irfh(:,:,1);
   irf(:,:,2)=irfh(:,:,2)+i*irfh(:,:,3);

   bin_vals = [0:floor((N/2-1))];
   fax_Hz = (bin_vals./(N/fs));%/ 100 from s to s aka from Hz to Hz

   fs_s = fax_Hz(2)-fax_Hz(1);
   tt=[0:GR_DWELL:GR_DWELL*(N-1)]';
   tt=tt+timeStart;

   figure(figNr+1)
```

```
for dir=1:3
    ft_gr(dir,:)=fft(gr(:,dir),N)./N; %complex
    up_ft_gr(dir,:)=interp1((fax_Hz),((ft_gr(dir,1:length(fax_Hz)))),[0:2:irf(1,end,1)],'linear');
    spl(dir,:)=(irf(dir,:,2).*(up_ft_gr(dir,:)./GR_DWELL));
end


for dir=1:3
    subplot(2,3,dir)
    plot(tt*1000,gr(:,dir),'color',sty{dir},'linewidth',2);
    xlabel('time in ms')
    ylabel('gradient strenght in T/m');
    title('gradient waveforms');
    hold on;

    subplot(2,3,dir+3)
    plot(fax_Hz, (abs((ft_gr(dir,1:length(fax_Hz))))),'color',sty{dir},'linewidth',3);
  %  axis([0, 8000, 0, max(max(abs(ft_gr(dir,1:length(fax_Hz))))),0.0001)]);

    xlabel('Frequency (Hz)')
    ylabel('Magnitude');
    title('Mag. spectrum (Hz)');
    hold on;
end

figure(figNr+2)
legendx={'IRF_y','A-weighting','g_y'};
legendy={'IRF_x','A-weighting','g_x'};
legendz={'IRF_z','A-weighting','g_z'};

for dir=1:3
    subplot(2,3,dir)
    axis([0, 8000, 0, max(max(abs(irf(dir,:,2))))]);
    mimi=min(min(min(abs(ft_gr(dir,1:length(fax_Hz)))),min(min(abs(irf(dir,:,2))))));
    mama=max(max(max(abs(ft_gr(dir,1:length(fax_Hz)))),max(max(abs(irf(dir,:,2))))));

    if first==0
        plot(irf(dir,:,1),abs(irf(dir,:,2)),'color',[120/256,120/256,120/256],'linewidth',1);
        hold on;
        plot(W/2/pi, 20*log10(abs(H)).*3,'color',[130/256,130/256,80/256],'linewidth',3);
    end
    hold on;
   plot(fax_Hz, abs(ft_gr(dir,1:length(fax_Hz)))./
(max(max(abs(ft_gr(dir,1:length(fax_Hz))))))*10,'color',sty{dir},'linewidth',3);


    xlabel('Frequency (Hz)')
    ylabel('Magnitude');

    xlabel('Frequency (Hz)')
    ylabel('Magnitude');
    title('Transfer functions and gradient spectra');
    hold on

    subplot(2,3,dir+3)
    plot(irf(1,:,1),(abs(spl(dir,:))),'color',sty{dir},'linewidth',3);
    hold on;

    xlabel('Frequency (Hz)')
    ylabel('Magnitude');
```

```
        title('IRF *g');
    end
end
```
